# Correction: Pagán, I.; García-Arenal, F. Cucumber Mosaic Virus-Induced Systemic Necrosis in *Arabidopsis thaliana*: Determinants and Role in Plant Defense. *Viruses* 2022, *14*, 2790

**DOI:** 10.3390/v15040852

**Published:** 2023-03-27

**Authors:** Israel Pagán, Fernando García-Arenal

**Affiliations:** Centro de Biotecnología y Genómica de Plantas UPM-INIA/CSIC and Departamento de Biotecnología-Biología Vegetal, E.T.S. Ingeniería Agronómica, Alimentaria y de Biosistemas, Universidad Politécnica de Madrid, 28045 Madrid, Spain; fernando.garciaarenal@upm.es

## Error in Figure

In the original publication [1], there was a mistake in Figure 4 as published. Panels A and B are identical and show the time course of CMV multiplication in Co-1 systemically infected rosette leaves. The corrected Figure 4 appears below. The authors apologize for any inconvenience caused and state that the scientific conclu-sions are unaffected. This correction was approved by the Academic Editor. The original publication has also been updated.

## Figures and Tables

**Figure 4:**
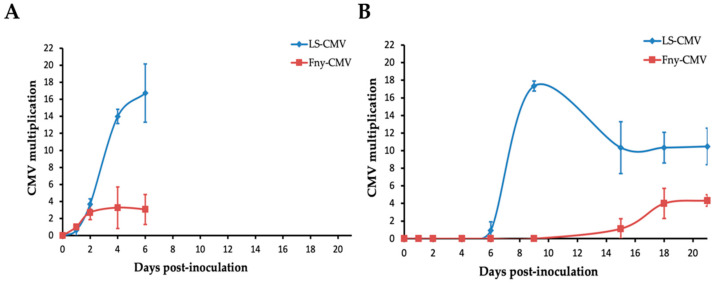
Time course of CMV multiplication in Co-1. CMV multiplication is estimated from the accumulation of viral RNA (mg/g fresh weight) of (**A**) inoculated rosette leaves and (**B**) systemically infected rosette leaves. Data for LS-CMV are represented as blue diamonds and data for Fny-CMV are represented as red squares. Data are mean ± standard error of three replicates per time point and virus.
