# Supplementary material for: Cucumber Mosaic Virus-Induced Systemic Necrosis in Arabidopsis thaliana: Determinants and Role in Plant Defense
Source: Viruses. 2022 Dec 14;14(12):2790. doi: 10.3390/v14122790 (PMC9783004; doi:10.3390/v14122790)
Supplement: Supplementary file 1 [file viruses-14-02790-s001.zip › viruses-2100348-supplementary.pdf]

Table S1. List of Arabidopsis ecotypes utilized in the screening for CMV-induced systemic necrosis.

Ecotype	ID[1]		Ecotype	ID[1]		Ecotype	ID[1]
**Ace-0**	9817		**Glo-1**	9848		**Rel-0**	9574
**Adc-5**	9513		**Gra-0**	9543		**Rev-0**	9576
**Adm-0**	9514		**Gud-3**	9849		**Ria-0**	9577
**Ang-0**	9519		**Hec-0**	9850		**Sac-0**	9578
**Ara-4**	9520		**Hom-4**	9546		**Sal-0**	9891
**Are-0**	9820		**Hum-2**	9549		**Ses-0**	9582
**Aru-0**	9821		**Jim-1**	9551		**Sfb-6**	9895
**Aul-0**	9822		**Lab-7**	9552		**Smt-1**	9897
**Bae-0**	9823		**Lam-0**	9855		**Sne-0**	9583
**Bar-1**	9521		**Leg-0**	9857		**Som-0**	9898
**Bea-0**	9522		**Loz-0**	9858		**Tam-0**	9586
**Bis-0**	9525		**Lro-0**	9859		**Tdc-0**	9587
**Boa-0**	9825		**Lso-0**	9554		**Tol-7**	9588
**Bor-0**	9826		**Lum-0**	9860		**Tor-1**	9589
**Bos-0**	9827		**Men-2**	9556		**Vae-2**	9592
**Cal-0**	9528		**Moa-0**	9557		**Val-0**	9903
**Cap-1**	9529		**Mon-5**	9559		**Vas-0**	9904
**Cas-0**	9831		**Mos**	9508		**Vav-0**	9511
**Cdc-3**	9531		**Mot-0**	9560		**Vaz-0**	9593
**Cdo-0**	9532		**Moz-0**	9870		**Vdm-0**	9594
**Cem-0**	9533		**Mun-0**	9561		**Vdt-0**	9595
**Cho-0**	9834		**Nac-0**	9871		**Ven-0**	9905
**Cmo-3**	9534		**Nog-17**	9564		**Ver-5**	9596
**Coa-0**	9507		**Oja-0**	9874		**Vig-1**	9597
**Coc-1**	9535		**Orb-10**	9565		**Vim-0**	9598
**Cor-0**	9536		**Ovi-1**	9875			
**Cot-0**	9838		**Pad-0**	9876			
**Coy-0**	9839		**Pal-0**	9567			
**Cum-1**	9537		**Pan-0**	9568			
**Dar-0**	9840		**Pds-1**	9569			
**Deh-1**	9539		**Pee-0**	9878			
**Ees-0**	9841		**Per-0**	9879			
**Esn-2**	9844		**Pie-0**	9881			
**Evs-0**	9845		**Pil-0**	9882			
**Ezc-2**	9846		**Prd-0**	9885			
**Fel-2**	9847		**Pro-0**	9571			
**Fun-0**	9542		**Rds-0**	9573			

[1] ID: Identification number in The Arabidopsis Information Resource.

Table S2. Primers used to sequence the *RCY1* gene in Co-1 plants.

Name	Sequence (5´-3´)	Location[2]	T_m (ºC)	Pair[3]
rppD[1]	CAATTTTGATTCCCTGCTTGCATCATCAAC	1-30	74.5	1
RCY1-1140fwd	TCTCTGAAGTGTTAACGAGCTC	1119-1140	60.2	2
RCY1-1560rev	ACGTCGAGTATCACCTACGA	1541-1560	59.7	1
RCY1-2580fwd	CTCGAAATGAAGGTGTTGG	2588-2606	60.7	3
RCY1-2880rev	TAGCATATGGATTACCATACG	2894-2914	56.6	2
RCY1-4200fwd	CATCTACCTTCTACTATGCGGA	4208-4229	59.8	4
RCY1-4500rev	AGAGTTTCAAAATTGCACC	4482-4500	56.7	3
RCY1-5160fwd	GTCATGTGGAGAGTCTCAACGA	5158-5180	58.3	5
RCY1-5400rev	CACAACATAACGATGCACTG	5368-5387	60.2	4
rppA[1]	ATTGTTCTCGTACTATTCGTTAGTCGTTAC	7480-7510	64.2	5

[1] Obtained from [31].

[2] Position of the *RCY1* nucleotide sequence in genotype C24 (Acc. N. AB082879).

[3] Order within the *RCY1* sequence of the primer pairs that amplify each of the five fragments spanning the complete nucleotide sequence of the gene.

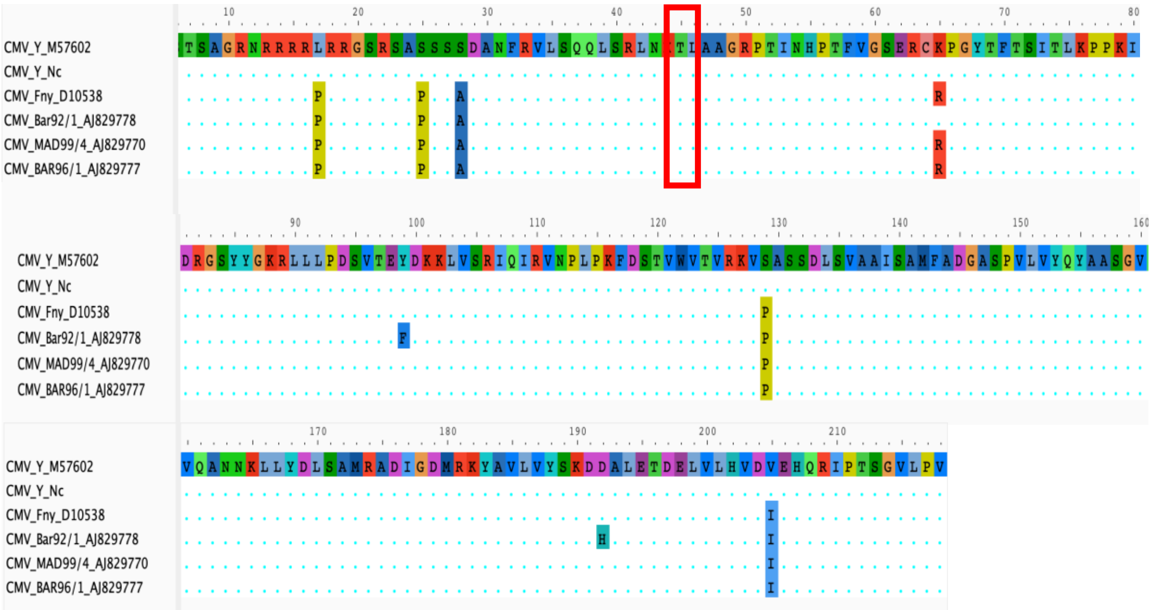

Figure S1. Comparison of the coat protein (CP) amino acid sequence of Y-CMV and the other subgroup I isolates used in this work. CMV-Y-Nc indicates our Y-CMV stock multiplied in *N. clevelandii*. Red rectangle indicates the position associated with systemic necrosis induced by Y-CMV in Arabidopsis.

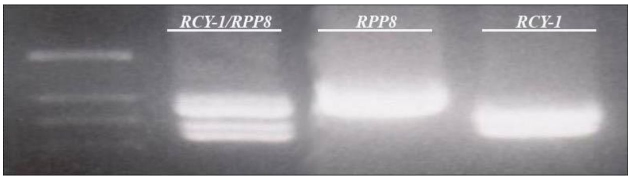

Figure S2. Detection of the *RCY1* and the *RPP8* alleles by PCR. Electrophoresis of PCR products in 2.5% agarose for plants *RCY1*/*RPP8* heterozygous (left), *RPP8* homozygous (center) and *RCY1* homozygous (right).

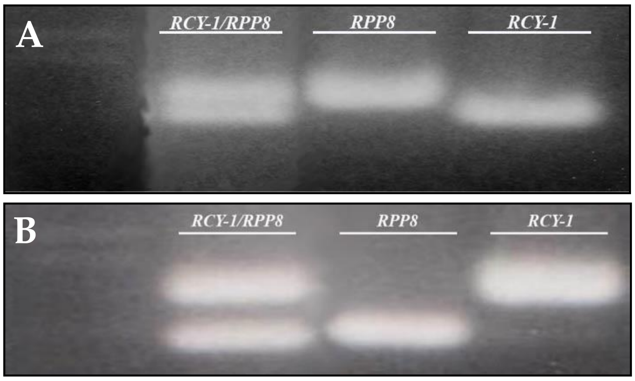

Figure S3. Detection of nga129 (A) and CIW9 (B) microsatellites by PCR. Electrophoresis of PCR products in 2.5% agarose for plants *RCY1*/*RPP8* heterozygous (left), *RPP8* homozygous (center) and *RCY1* homozygous (right).
